# Proteomic characterization of *Aspergillus fumigatus* – host interactions using the *ex-vivo* pig lung (EVPL) model

**DOI:** 10.1080/21505594.2025.2530675

**Published:** 2025-07-15

**Authors:** Aaron Curtis, Freya Harrison, Kevin Kavanagh

**Affiliations:** a Department of Biology, Maynooth University, Maynooth, Co. Kildare, Ireland; b School of Life Sciences, University of Warwick, Coventry, UK

**Keywords:** *Aspergillus*, *ex vivo*, infection, porcine, proteomics

## Abstract

*Aspergillus fumigatus* is an opportunistic fungal pathogen of the human airway that can cause a variety of chronic infections, typically in the context of pre-existing lung damage. The interaction of *A. fumigatus* with *ex-vivo* pig lung (EVPL) samples was characterized at the proteomic level to provide insights into how the fungus may interact with pulmonary tissue *in vivo*. This model has many advantages, because pigs share 90% immunological homology with humans and display many anatomical similarities. EVPL also retains resident immune cells, has richer cellular complexity compared to *in-vitro* models, and has a microbiome. Label-free quantitative proteomic analysis identified the metabolism and development of *A. fumigatus* on the EVPL alveolar sections; at 48 h, there was an increased abundance of proteins associated with carbon metabolism (e.g. malate dehydrogenase (+8.2 fold increase)), and amino acid metabolism and biosynthesis (e.g. 5-methyltetrahydropteroyltriglutamate – homocysteine S-methyltransferase, (+5.04 fold)) at 72 h. Porcine tissue remained responsive to the pathogen with proteins that increased in abundance associated with innate immune recruitment (e.g. protein S100-A8 (+28.5 fold) and protein S100-A9 (calgranulin-B) (+7.25 fold)) at 24 h, while proteins associated with neutrophil degranulation (e.g. elastase, neutrophil (−2.74 fold)) decreased in abundance. At 96 h, the infected tissue demonstrated enhanced abundance of fibrotic markers (e.g. fibrillin 1, collagen type IV alpha 1 chain, and alpha 2 chain, increased by + 16.44, +15.42 and + 11.95 fold, respectively). These results validate the use of this model for studying pathogen-host interactions and highlight how *A. fumigatus* interacts with pulmonary tissue during colonization.

## Introduction

The filamentous fungus *Aspergillus fumigatus* is an environmental saprophyte and opportunistic pathogen in the human airway [1]. Many infections caused by *A. fumigatus* are chronic, with chronic pulmonary aspergillosis affecting approximately 1.8 million patients annually, with an 18.5% mortality rate. Fungal asthma, which has been partially attributed to *Aspergillus* exposure, affects an estimated 11.5 million people, resulting in 46,000 deaths annually [2]. A common underlying factor in many chronic infections is pre-existing lung damage, inflammation, and cavitation caused by COPD, pulmonary tuberculosis, cystic fibrosis (CF), bronchiectasis, thoracic radiotherapy, and allergic bronchopulmonary aspergillosis [3]. The initial attachment and colonization of the lung by fungi is facilitated by interactions with the components of the host extracellular matrix and basal lamina [4]. Various conditions, including CF and asthma, result in the deposition of collagen and fibronectin, which can serve as substrates for fungal conidia adhesion [5,6]. The complex interplay between *A. fumigatus* and its host has been partially elucidated through clinical studies and the utilization of diverse *in vitro* and *in vivo* model systems [7]. Despite this, these model systems frequently fail to fully demonstrate the intricacies of human airway infections and, as a result, often fail to achieve the clinical phenotypes observed in the clinic [8,9]. Murine models have been the focus of the majority of aspergillosis *in vivo* studies because of the observed similarities in physiology and pathology in addition to genomic similarities [10] however, they demonstrate considerable heterogeneity in their susceptibility to infection [11]. In addition, despite the fact that rodents and human lungs contain many of the same cell types, the anatomy of the lung varies. Human lungs contain basal cells throughout the trachea and bronchi, whereas murine basal cells are only found in the trachea. There is also no evidence that human lungs possess a bronchioalveolar stem cells population as the majority of cells in the human proximal airway are multi-ciliated cells, whereas club cells are more abundant in rodents [12]. Goblet cells are prevalent in the proximal human airway, but they primarily appear in mice following injury [13]. The chemical composition of murine lung tissue and airway surface liquid is also distinct from that of human airways [14]. The human lung metabolome was distinguishable from the murine metabolome in terms of trimethylamine *N*-oxide, betaine, carnitine, and glycerophosphocholine, which are present in mice but not in the human lung. In addition, fatty acid concentrations are significantly higher in rodent lungs than in human lungs. Acetate, asparagine, glutamate, lactate, lysine, myo-inositol, syllo-inositol, and valine concentrations were considerably lower in murine lungs than in human lungs. The metabolome of pig lungs demonstrated a similar composition to that of humans, with the main difference being the concentration of some components [15]. This difference may contribute to the failure of murine models to replicate pivotal aspects of human diseases and infections such as aberrant abscess formation which occurs in murine lungs during *Staphylococcus aureus* infection [16], but is rarely observed in human CF patients [17]. Clinically relevant lumen colonization with preferential localization as multicellular aggregates in mucus was observed in bronchiolar pig lung sections, which better recapitulates what is observed in human biopsies [18].

Many alternative systems have been employed to study fungal pathogenesis, including insect mini models such as *Galleria mellonella* larvae [19], cell culture [20], and organoid models [21*]. G. mellonella* is the most utilized insect model for fungal infection studies, and larvae are easy to inoculate and exhibit a dynamic response to pathogens, comparable to the innate immune response in humans [22]. In addition, *G. mellonella* larvae infected with fungal pathogens show structures characteristic of human infection, including granuloma development during *A. fumigatus* infection [23] and grain formation during *Madurella mycetomatis* infection [24]. The *Galleria* infection model has demonstrated excellent correlation with experiments that assessed the virulence of *Candida albicans* and *Pseudomonas aeruginosa* in mice [25,26]. The major limitation of the use of *G. mellonella* larvae is the lack of an adaptive immune response and organs, such as the lungs, which limits the model to the study of invasive and bloodstream infections [27].

Complex tissue models and organoids show a considerable increase in utility compared with 2D cell culture studies [28,29]. Although the complexity of organoids has increased, they are unable to accurately reproduce the pathological characteristics of the human lung, including angioinvasion. The human lung is composed of over 40 different cell types [30], and current hPSC-derived lung organoids remain incomplete because they lack many lung components, such as vasculature and complex immune cell diversity [31]. In addition to these limitations organoid models require specialist tissue culture facilities and techniques and can be expensive; therefore, they are a relatively low throughput approach for infection studies [32]. Lung damage is a common precursor to many forms of aspergillosis; as many three-dimensional organoids are derived from human pluripotent stem cells they tend to resemble the foetal lung, they may not be suitable to mimic these conditions [31].

Pig lung models have been developed in an attempt to overcome many of these limitations owing to their immunological and physiological similarities with humans, and the microbiome of healthy pig lungs shows a similar phylum distribution to that found in human lungs [33,34]. The *ex-vivo* lung perfusion model (EVLP) demonstrated pathogen- and virulence factor-specific responses to *Klebsiella pneumoniae* infection in a whole lung system with whole blood perfusion and ventilation over 4 hours post-mortem [35]. Despite the successful implementation of this model, it requires a large amount of space and specialized equipment and skills. The *ex-vivo* pig lung model (EVPL) offers a high-throughput, low-cost, and ethical model that closely mimics the lung environment [36]. Lungs can be obtained from pigs slaughtered for commercial meat production from butchers or abattoirs and since little or no lung tissue is used in food production, lungs are classified as a waste product whose use does not raise ethical questions [36]. This method was first developed by Williams & Gallagher, who collected lungs from commercial abattoirs and used tissue sections to study *Mycoplasma* infection [37,38]. This model was then optimized to mimic CF airways and used to study bacterial pathogenicity [39] and antibiotic tolerance [18,40]. This system involves the excision of multiple sections of the bronchiolar or alveolar tissue from the lungs of a single donor. These can be inoculated with pathogens and various endpoints can be examined. This model has demonstrated strain-specific virulence differences, including quorum sensing-deficient mutants of *Pseudomonas aeruginosa* demonstrating reduced damage to alveolar tissue [36]. It has also been demonstrated that the EVPL model shows *in vivo*-like aspects of *P. aeruginosa* gene expression and that the pathogen forms a biofilm using known *in vivo* pathways required during *in vivo* infection, resulting in the formation of clinically realistic structures not seen in other *in vitro* studies [40,41].

Once inhaled, *Aspergillus* conidia that are not cleared by mucociliary elevators encounter epithelial cells or alveolar macrophages [42]. These conidia are often deposited in the bronchioles and alveolar spaces because of the small size of the fungal conidia (2–3 μm), which is ideal for deep infiltration into alveolar spaces [7]. We combined alveolar sections of pig lung tissue with standard tissue culture medium to assess the tractability of this model for working with fungi and to mimic human tissue in the absence of any underlying conditions that radically alters lung chemistry [43]. We examined the development of *A. fumigatus* in this physiologically sustainable and ethical model to gain insight into how the host and the pathogen respond to each other during the early stages of fungal infection.

## Materials and methods

### *Aspergillus fumigatus* culture conditions and conidial preparation

*Aspergillus fumigatus* ATCC 26,933 was cultured for 72 h at 37°C on malt extract agar (MEA) (Oxoid, Basingstoke, UK) following point inoculation. The conidia were harvested by washing with phosphate-buffered saline supplemented with 0.1% (*v*/*v*) Tween-20 (PBS-T), and the suspension was washed three times with PBS. Conidia were enumerated using a haemocytometer and diluted to a final concentration of 1x10^7^ conidia/ml.

### Preparation of ex-vivo pig lung sections

Alveolar tissue sections were prepared as described [36], with some modifications. Whole lungs from four individual pigs with attached tracheae were collected from a local abattoir within an hour of slaughter and were transported on ice to the university. The pleural surface of the caudal lobe was sterilized by briefly touching it with a hot palette knife, and ~5 mm-deep strips of alveolar tissue were cut from the sterilized surface using a mounted razor blade. These were washed in a 50/50 mixture of RPMI 1640 (Gibco) and Dulbecco’s modified Eagle medium [DMEM] (Gibco) supplemented with 10 µg/ml amphotericin B to remove any environmental fungi. The strips were cut to 125 mm^3^ explants and washed twice in a 50/50 mix of RPMI 1640 and Dulbecco’s modified Eagle medium (DMEM) supplemented with 50 µg/mL ampicillin to reduce bacterial load (components of the pig lung microbiome or environmental contaminants). The tissue sections were washed in the RPMI/DMEM mixture and UV sterilized for 5 min before being placed on a pad of 400 µL RPMI/DMEM solidified with 0.8% agarose in a 24 well tissue culture plate. The sections were inoculated using a 25 G needle dipped into the prepared *A. fumigatus* conidia suspension (standardized to 1 × 10^7^/ml) and used to inoculate the surface of the section to deposit fungal spores. The sections were suspended in 500 μl RPMI/DMEM. The sections were covered with a breathable membrane (Breathe-easy sealing membrane, Diversified Biotech, USA) before incubation in a 6% CO_2_ incubator at 37°C to match the physiological carbon dioxide levels observed in the alveoli [44]. Sterilized solidified malt extract agar (MEA, Oxoid) was aseptically cut into the same dimensions (5 × 5 × 5 mm) as EVPL tissue and used as a control for fungal growth.

### Quantification of fungal burden

Infected tissue sections were removed from the 24 well plate with sterilized forceps at 24-hour intervals and washed in sterile PBS prior to transfer into 2 ml Eppendorf tubes with 1 ml of lysis buffer and a 3 mm chrome stainless steel ball bearing. The tissue was homogenized using a tissue lyser (TissueLyser II, Qiagen, Germany) at 30.0 frequency 1/s for 40 seconds. Lysate (100 µL) was diluted and plated onto MEA plates in triplicate and incubated at 37°C overnight. The colony-forming units (CFU) were enumerated. The average number of colonies resulting from each treatment was determined and a 2-way ANOVA analysis was performed. Figures were generated using Prism v8.01. Images of infected tissue were viewed at 40x magnification using a brightfield microscope (Olympus CH20).

### Proteomic extraction from infected tissue sections

Infected and control EVPL sections were removed from 24 well plates and washed with PBS. The sections were transferred to 2 ml Eppendorf tubes with 1 ml of lysis buffer (8 M urea, 2 M thiourea, and 0.1 M Tris-HCl (pH 8.0) dissolved in HPLC-grade ddH_2_O), supplemented with protease inhibitors (aprotinin, leupeptin, pepstatin A (10 µg/mL), and phenylmethylsulfonyl fluoride (PMSF) (1 mM/mL)). Tissue sections were homogenized as previously described. The lysates were sonicated (Bandelin Senopuls) three times for 10 s at 50% power. The cell lysate was centrifuged (Eppendorf Centrifuge 5418) for 8 min at 14,500× *g* to pellet cellular debris. Lysate (200 µL) was precipitated with acetone for 18 hours at −20°C. Samples were subjected to centrifugation at 14,500× *g* for 10 min to pellet proteins, acetone was removed, and the pellet was resuspended in 50 µL sample resuspension buffer (8 M urea, 2 M thiourea, and 0.1 M Tris-HCl (pH 8.0) dissolved in HPLC-grade ddH_2_O), of which 40 µL was digested. A 2 µL aliquot was removed from each sample prior to digestion for quantification using the Qubit quantification system (Invitrogen, Waltham, MA, USA). Ammonium bicarbonate (250 µL, 50 mM) was added to 40 µL, which was subjected to reduction via the addition of 2 µL of 0.5 M dithiothreitol and incubated at 56°C for 20 min, followed by alkylation with 0.55 M iodoacetamide at room temperature in the dark for 15 min. Proteins were digested by adding 2 µL of sequence-grade trypsin (Promega) (0.5 µg/µL), supplemented with 2 µL of Protease Max Surfactant Trypsin Enhancer (Promega) (1% *w*/*v*), and incubated at 37°C for 18 h. Digestion was quenched by the addition of 2 µL of trifluoroacetic acid (TFA) and incubated at room temperature for 5 min. The samples were centrifuged at 14,500× *g* for 10 min prior to clean-up using C18 spin columns (Pierce). The eluted peptides were dried using a SpeedyVac concentrator (Thermo Scientific (Waltham, MA, USA) Savant DNA120) and resuspended in 2% (*v*/*v*) acetonitrile and 0.1% (*v*/*v*) formic acid to yield a final concentration of 375ng/µL aided by sonication in a water bath for 5 min. The samples were centrifuged to pellet debris at 14,500× *g* for 5 min, and 2 µL of each sample was loaded onto the mass spectrometer.

### Mass spectrometry

Purified peptide extracts (2 μL containing 750 ng protein) were loaded onto a Dionex UltiMate 3000 RSLCnano system equipped with an Easy-Spray C18 HPLC column (Thermoscientific) connected to a Q Exactive Plus Hybrid Quadrupole-orbitrap mass spectrometer (Thermo Fisher Scientific, Waltham, MA, USA) and eluted at a flow rate of 0.3 µL per minute using a reverse phase 133 minute gradient. A scan range of 200–1600 M/Z with a resolution of 70,000 was used. The top 15 ions were selected from each MS scan with an isolation window of 2 M/Z for fragmentation and an MS/MS scan in the range of 200–2000 M/Z with a resolution of 17,500. Raw MS/MS data files were processed using the Andromeda search engine in MaxQuant software v.1.6.3.4 110 using a *Neosartorya fumigata* reference proteome obtained from a UniProt-SWISS-PROT [45] database to identify proteins (9647 entries, downloaded July 2022) or the *Sus scorfa* reference proteome (46,174 entries, downloaded December 2023) respectively.

### Data analysis

Proteomic data analysis was performed as described [46], with some modifications. Perseus v.1.6.15.0, was used for data analysis, processing, and visualization. Normalized LFQ intensity values were used to quantitatively measure protein abundance. The generated data matrix was filtered to remove contaminants. LFQ intensity values were log_2_-transformed, and each sample was assigned to its corresponding group matching the time point at which they were collected. Proteins that were not found in all replicates in at least one group were omitted from further analysis. A data-imputation step was conducted to replace missing values with values that simulate signals of low-abundance proteins chosen randomly from a distribution specified by a downshift of 1.8 times the mean standard deviation of all measured values and a width of 0.3 times this standard deviation. Principal component analysis (PCA) was performed using normalized intensity values. The identified proteins were then defined using a Perseus annotation file to assign extract terms for biological process, molecular function, and Kyoto Encyclopaedia of Genes and Genomes (KEGG) names. To visualize the differences between two samples, pairwise Student’s *t*-tests were performed using a cut-off of *p* < 0.05 on the post-imputation dataset. Volcano plots were generated by plotting the log_2_ fold change on the x-axis against the log *p*-values on the y-axis for each pairwise comparison. Statistically significant and differentially abundant (SSDA) proteins (ANOVA, *p* < 0.05) with a relative fold change greater than ± 1.5 were retained for analysis. SSDA proteins were z-score-normalized and then used for hierarchical clustering to produce a heat map. Identified SSDAs were then assessed using Uniprot codes generated by Perseus, and pathway analysis was performed using ShinyGO [47] to gain insights into their roles within the cells. The mass spectrometry proteomics data were deposited in the ProteomeXchange Consortium via the PRIDE [48] partner repository with the dataset identifier PXD060389.

## Results

### Confirmation of *Aspergillus fumigatus* growth on EVPL tissue

**Figure 1.**
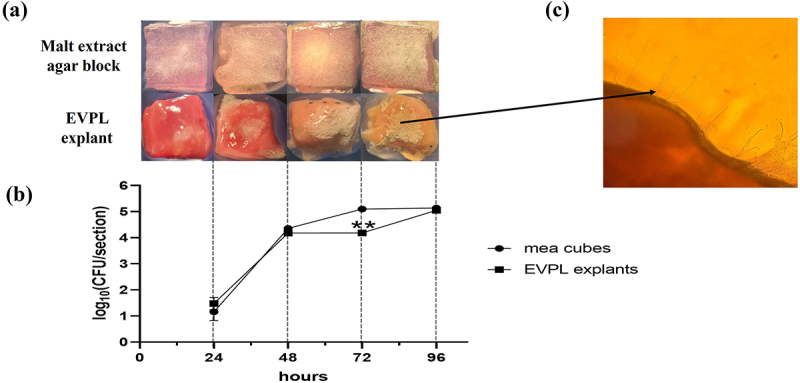
Phenotypic analysis and confirmation of fungal growth on agar and *ex-vivo* pig lung explants. (a) Representative image of sections at each 24 hour interval imaged via dissection microscope. (b) Graph of fungal burden calculated from agar and EVPL explants showing a significant decrease in CFU at 72 hours (*p* = 0.002) calculated by two-way ANOVA. (c) Microscopy image of hyphal growth on an EVPL section at 40x magnification.

MEA cubes (*n* = 3) and EVPL explants (*n* = 3) for each time point were inoculated with *A. fumigatus* conidia and incubated at 37°C and 6% CO_2_. Visual inspection of agar cubes revealed the growth of *A. fumigatus* at 24 h and this increased until 96 h when conidiation was evident (Figure 1a). In contrast, there was a small amount of visible *A. fumigatus* growth on the EVPL explants at 24 h, but more extensive fungal growth was evident at 48 h. After 96 h, a large *A. fumigatus* colony was observed on the surface. Fungal growth was also assessed by quantifying the number of colony-forming units (CFU) per treatment (Figure 1b). The growth on the MEA cubes and EVPL explants was initially comparable, but there was a significantly (*p* = 0.002) lower fungal CFU at 72 h in the EVPL relative to the MEA cubes with 1.2 × 10^5^ CFU on the MEA cube compared to 1.5 × 10^4^ CFU detected in the EVPL section. The growth on the EVPL was comparable to that on the MEA cubes at 96 h, with approximately 1.4 × 10^5^ CFU/section (Figure 1). *A. fumigatus* hyphae were visible in the infected tissue (Figure 1c).

### Proteomic analysis of alterations in *A. fumigatus* proteome during growth on EVPL tissue

**Figure 2.**
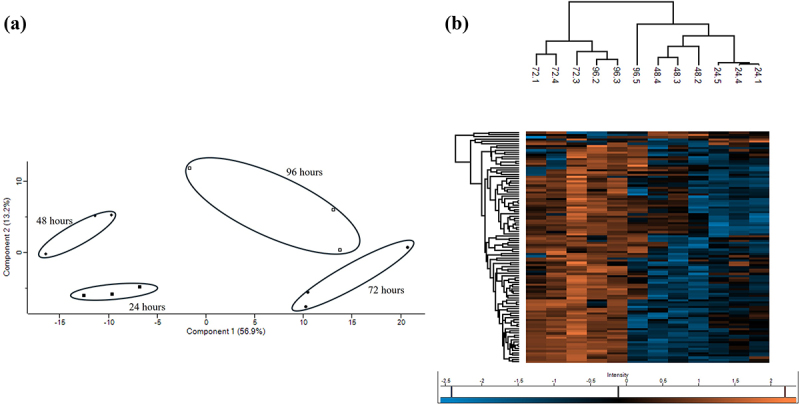
(a) Principalal component analysis of *A. fumigatus* proteins at 24, 48,72 and 96 hours demonstrating good separation in the proteome at each timepoint. (b) Heatmap generated through two-way unsupervised hierarchical clustering of the median protein expression values of all statistically significant differentially abundant proteins.

Quantitative proteomic analysis was used to characterize the changes in the fungal proteome during colonization of EVPL explants. The fungal proteomes (*n* = 3) at each time point were characterized, and these were well separated, as seen in the PCA and heatmap (Figure 2a,b). Changes in the relative abundance of fungal proteins were compared with those of the *A. fumigatus* proteome at 24 h. At 48 h post-infection, 15 proteins were significantly increased in abundance and 17 were significantly decreased (Table S1). Many proteins that showed an increase in abundance were associated with carbon metabolism, including glyceraldehyde-3-phosphate dehydrogenase (+10.90 fold). Glyceraldehyde-3-phosphate dehydrogenase expression is associated with conidial germination, is expressed on the hyphal surface, and has been speculated to aid fungal adherence to host tissue [49]. Phosphoglycerate kinase (+3.60 fold) is involved in carbon metabolism but is also part of the *Afpes1* NRPS cluster involved in fumigaclavine C biosynthesis [50]. There was also increased abundance of fungal allergens, such as large ribosomal subunit protein P2 (60S acidic ribosomal protein P2) (AfP2) (allergen Asp f 8) (+6.99 fold) and superoxide dismutase [Mn], mitochondrial (allergen Asp f 6) (+4.40 fold) which may be involved in inducing an immune response within the tissue [51]. Proteins decreased in abundance at 48 h, including malate synthase and dihydrolipoyl dehydrogenase (−3.47 and −3.86 fold, respectively), which are involved in alternative metabolic processes (Table S1). Gene enrichment analysis indicated that carbon metabolism was enhanced, and amino acid metabolism was decreased at this timepoint (Figure S1).

At 72 h post-infection, 66 proteins were significantly increased in abundance relative to that at 24 h, and no proteins were significantly decreased (Table S2). Many proteins that increased in abundance were associated with amino acid biosynthesis and metabolic processes, including aconitate hydratase, mitochondria (+6.26 fold), 5-methyltetrahydropteroyltriglutamate – homocysteine S-methyltransferase (+5.04 fold), 4-aminobutyrate aminotransferase (+2.80 fold), and acetohydroxy-acid reductoisomerase (+1.97 fold), which have also been implicated in fungal iron homoeostasis [52–54]. A significant increase in abundance was observed for the 14–3–3 family protein ArtA, putative (+35.63 fold). ArtA is a regulatory protein associated with the response to oxidative stress [55]. There was also a significant increase in the abundance of large ribosomal subunit protein P1 (60S acidic ribosomal protein P1) (AfP1) (+19.17 fold) and large ribosomal subunit protein P2 (60S acidic ribosomal protein P2) (AfP2) (allergen Asp f 8) (+18.42 fold) indicating enhanced translation and protein biosynthesis (Table S2). Gene enrichment analysis indicated elevated biosynthesis and metabolism of amino acids at this time (Figure S2).

At 96 h post-infection, 44 proteins were significantly increased in abundance relative to that at 24 h, and two proteins were significantly decreased (Table S3). Proteins increased in abundance, including asp-haemolysin (+6.49 fold), and dipeptidyl-peptidase 5 (+11.67 fold) at 72 hours and + 9.61 fold at 96 h, and are known to be induced in murine infection [56,57]. Thioredoxin reductase gliT, involved in self-protection during gliotoxin production and oxidative stress mitigation [58], was increased (+5.97 fold). Short-chain dehydrogenase, which was previously shown to be induced by gliotoxin exposure [59], increased by + 9.21 fold at 72 hours and + 17.37 fold at 96 h. Woronin body major protein hexA, involved in physical stress meditation through septal pore formation and virulence [60], increased in abundance at 72 (+11.73 fold) and 96 (+9.86 fold) hours, indicating the occurrence of host-induced damage. Proteins that decreased in abundance at 96 h were triosephosphate isomerase (−2.09 fold), involved in glucose metabolism, and phytanoyl-CoA dioxygenase family protein (−2.31 fold), and similar proteins are known to be involved in the production of fungal toxins, including verruculogen [50], typically expressed during the early stages of infection. Gene enrichment analysis (Figure S3) highlighted the increased expression of proteins associated with ascorbate and aldarate metabolism, beta-alanine metabolism, fatty acid degradation, glycolysis/gluconeogenesis, tryptophan metabolism, and degradation of valine, leucine, and isoleucine, all of which have been demonstrated to be enhanced following *A. fumigatus* exposure to human dendritic cells [61].

Some *A. fumigatus* proteins were detected at two or more sampling timepoints (Table 1). These proteins including the suspected allergen 60S ribosomal protein L12 [62] detected at 48 and 72 hours post infection. Other proteins were consistently increased in abundance at all three time points and included cyanovirin-N domain-containing protein, which increased + 13.14-, +18.00 and + 23.01 fold at 48, 72, and 96 h, respectively. This protein belongs to a family of highly conserved proteins that are known to bind strongly to mannose, potentially enhancing fungal attachment to the host [63] and affecting the morphology of PBMCs [64]. Mannose-dependent c type lectin interactions have been shown to be impeded by cyanovirin-N [65] and as such consistent expression of this protein could indicate its role in immune evasion by *A. fumigatus*.

**Table 1.** Statistically significant and differentially abundant *Aspergillus fumigatus* proteins associated with virulence or involvement in eliciting an immunological response within the host detected at two or more timepoints relative to a 24-hour sample.

protein ID	Protein name	48 hours	72 hours	96 hours
Superoxide dismutase [Mn], mitochondrial (EC 1.15.1.1) (allergen Asp f 6)	Q92450	4.40	9.29	N/A
14–3–3 family protein ArtA, putative	Q4WI29	−2.14	35.63	N/A
60S ribosomal protein L12	Q4WK81	−3.15	3.15	N/A
Malate synthase (EC 2.3.3.9)	Q4WD53	−3.47	3.47	N/A
Phytanoyl-CoA dioxygenase family protein	Q4WZT3	−4.03	N/A	−2.31
Acyl CoA binding protein family	Q4X164	N/A	13.93	11.24
Woronin body major protein hexA	Q4WUL0	N/A	11.73	9.86
Dipeptidyl-peptidase 5 (EC 3.4.14.-) (Dipeptidyl-peptidase V) (DPP V) (DppV)	P0C959	N/A	11.67	9.61
Short chain dehydrogenase, putative (EC 1.-.-.-)	Q4WPB8	N/A	9.21	17.37
Thioredoxin	Q4WV97	N/A	3.21	5.26
Cyanovirin-N domain-containing protein	Q4WKJ1	13.14	18.00	23.01
Glyceraldehyde-3-phosphate dehydrogenase (EC 1.2.1.12)	Q4WE70	10.90	30.96	33.92
Malate dehydrogenase (EC 1.1.1.37)	Q4WE70	8.20	23.43	11.52
Large ribosomal subunit protein P2 (60S acidic ribosomal protein P2) (AfP2) (allergen Asp f 8)	Q9UUZ6	6.99	18.42	12.60
Enolase (EC 4.2.1.11) (2-phospho-D-glycerate hydro-lyase) (2-phosphoglycerate dehydratase) (allergen Asp f 22)	Q96X30	6.08	12.98	13.54
Methyltransferase psoC (EC 2.1.1.-) (Pseurotin biosynthesis protein C)	Q4WB00	5.63	6.80	6.77
Alanine transaminase (EC 2.6.1.2)	Q4WN34	3.81	8.60	4.92
G-protein complex beta subunit CpcB	Q4WQK8	3.73	6.12	4.40

The G-protein complex beta subunit CpcB was also detected at all time points (+3.73-, +6.12, and + 4.40 fold, at 48, 72, and 96 h, respectively) and plays an essential role in cellular growth, spore germination, and conidiation [66]. Methyltransferase psoC increased + 5.63-, +6.80, and + 6.77 fold, respectively, and is involved in the synthesis of pseurotin A, which can suppress RankL-induced oxidative stress [67]. Enolase was increased by + 6.08-, +12.98, and + 13.54 fold, respectively, and is involved in glycolysis, it has been identified as a potential inhibitor of the human complement cascade by binding to Factor H, FHL-1, C4BP, and plasminogen [68].

### Characterisation of proteomic alterations in EVPL tissue during *A. fumigatus* colonisation

**Figure 3.**
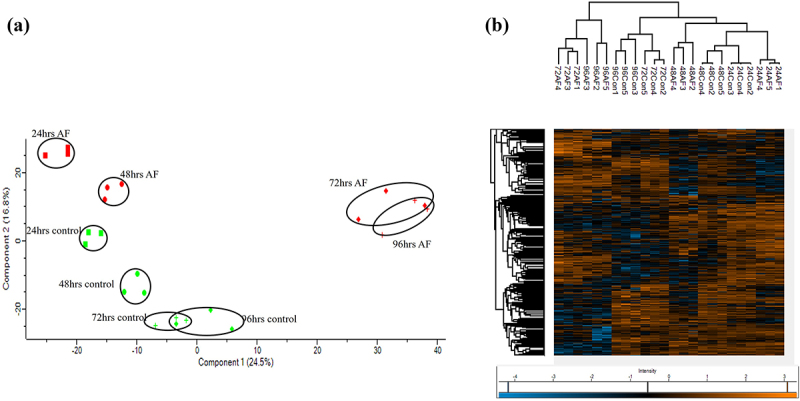
(a) Principalal component analysis of *Sus scrofa* proteins at 24, 48,72 and 96 hours demonstrating good separation in the proteome at each timepoint. (b) Heatmap generated through two-way unsupervised hierarchical clustering of the median protein expression values of all statistically significant differentially abundant proteins.

**Figure 4.**
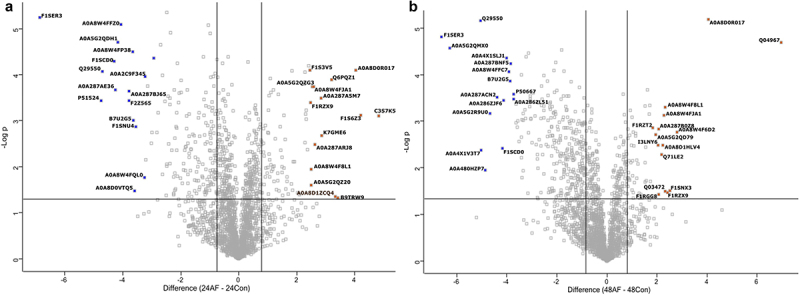
Volcano plots showing the distribution of statistically significant and differentially abundant (SSDA) proteins which have a −log (*p*-value) > 1.3 and difference ± 0.58. (a) *Sus scrofa* infected lung explants with *A. fumigatus* compared to uninfected lung explants at 24 hours and (b) *Sus scrofa* infected lung explants with *A. fumigatus* compared to uninfected lung explants at 48 hours.

The pig proteome (*n* = 3) at each time point during infection was characterized, and these were well separated, with clear differences between infected and uninfected tissue, as shown in the PCA and heatmap with some overlap observed at later time points (Figure 3a,b). The early response to infection elicited many changes in the porcine proteome, indicating a dynamic response to *A. fumigatus* (Figure 4a,b). At 24 h post-infection, 123 proteins were significantly increased in abundance in infected EVPL tissue, and 212 were decreased relative to uninfected tissue (Table S4). Protein S100-A8 and protein S100-A9 (calgranulin-B) were increased in abundance (+28.25 and + 7.25 fold, respectively), and S100A8/A9 plays a critical role in modulating the proinflammatory response by stimulating leukocyte recruitment and inducing cytokine secretion [69–71]. Costar family protein ABRACL was increased in abundance in the infected tissue (+18.58 fold) and is associated with immune cell infiltration [72]. Carbonic anhydrase 4 was also increased + 10.09 fold and was expressed on IL-5-activated eosinophils, indicating that an allergic response could be elicited at this timepoint [73]. Tetraspanin was increased in abundance + 5.49 fold in the infected tissue and is involved in forming functional interactions with prominent leukocyte receptors including MHC molecules [74,75]. Gene enrichment analysis indicated enrichment I antigen processing and presentation, particularly through MHC class II, phagosome, and neutrophil extracellular trap formation, indicating that an active immune response was induced within the infected tissue [76,77] (Figure S4). Proteins decreased in abundance, including pulmonary surfactant-associated protein A1 isoform X2 (−115.77 fold) which is integral to preventing airway collapse and is known to bind to fungal carbohydrates, enhancing fungal phagocytosis [78]. The levels of surfactant protein A are decreased in the lungs of patients with CF, acute respiratory distress syndrome, and other chronic lung diseases [79]. This can be attributed to neutrophilic recruitment factors including cathepsins which can degrade pulmonary surfactant A [80]. Dipeptidyl peptidase 1 (cathepsin C) and cathepsin S were increased 1.53 and 1.71 fold respectively at 24 hours post infection. Cathepsin C has been demonstrated to be involved in inflammation and pathogenesis in both acute and chronic disease [81]. Cathepsin S has been demonstrated to directly cleave surfactant protein A and has been implicated in lung injuries and tissue remodeling associated with CF [82]. Prophenin and tritrpticin precursor (C6) (−26.95 fold), antibacterial protein (cathelicidin antimicrobial peptide preproprotein) (−18.02 fold), lipocalin 2 (−13.68 fold), proteinase 3 (−12.00 fold) and elastase, neutrophil expressed (−2.74 fold) were decreased in abundance in the infected tissue and are involved in neutrophil activity and degranulation [83–87]. Gene enrichment analysis (Figure S4) also highlighted decreases in proteins associated with the citrate cycle and amino acid degradation indicating the infected tissue is less metabolically active which is similar to that observed in murine models of invasive pulmonary Aspergillosis [88].

At 48 h, 88 proteins were increased in abundance and 351 proteins were decreased in abundance in the infected tissue (Table S5). Heat shock 70 kDa protein 6 was increased by + 125.30 fold, and this protein is induced during stress and has been associated with the infiltration of immune cells [89,90]. Importin subunits alpha, KPNA3 and KPNA4 were increased by + 6.92 and + 4.99 fold, respectively, and are essential for TNF-alpha-stimulated NF-kappaB p50/p65 heterodimer translocation into the nucleus [91]. KPNA4 expression was also positively correlated with the infiltration of CD8+ T cells, B cells, dendritic cells, CD4+ T cells, neutrophils and macrophages [92]. Marginal zone B-and B1-cell-specific protein (MZB1) and Histone H3.3 also increased in abundance (+4.10 and + 4.52 fold, respectively), and these proteins play a role in the humoral immune response and are involved in differentiation to plasma cells. Histone H3.3 is involved in maintaining B-cell and CD8^+^ T-cell function and prevents premature haematopoietic stem cell exhaustion and differentiation into granulocyte-macrophage progenitors [93]. The first markers of pulmonary fibrosis were detected at this timepoint, including indolethylamine N-methyltransferase (+4.17 fold), associated with myofibroblast formation [94,95], nestin (+4.17 fold), which is expressed in myofibroblasts and has a pro-fibrotic function by facilitating Rab11-dependent recycling of TGF-β receptor I [96]. TGF-β was significantly enhanced at 24 h post-infection (+2.70 fold).

Proteins decreased in abundance at 48 h, including histone H2A (−78.18 fold) which plays a role in double-strand break repair [97]. FLII actin remodeling protein was also decreased −29.04 fold, and knockouts of this protein have been associated with increased numbers of myofibroblasts [98] (Table S5). There is also evidence of disruption of the epithelial tight junction with reduced abundance of junctional adhesion molecule A (−3.54 fold) and claudin 18 (−2.97 fold) both integral to epithelial integrity [99,100]. Complement factor B (C3/C5 convertase) and Complement C3 decreased in abundance (−2.58 and −2.04 fold, respectively), indicating that immune evasion induced by the fungus could be occurring [68]. Other known complement evasion mechanisms observed in *A. fumigatus* include recruitment of the human plasma regulators factor H, FHL-1, C4BP, and plasminogen and pentraxin-3 and ficolin-2 [101], none of which was significantly altered in our analysis. The secretion of proteases alp1 and mep1 has also been shown to degrade or cleave complement factors [102]. These were also not detected in either the host or pathogen analysis presented here. This implies that enolase is a potent inhibitor of this cascade or other factors yet to be identified are involved in the evasion observed in this study. Gene enrichment analysis (Figure S5) demonstrated a response to fungal infection with enriched pathways, including leukocyte transendothelial migration and chemokine signaling pathways, while metabolism was decreased (i.e. 2-oxocarboxylic acid metabolism and propanoate metabolism). Elevated levels of propanoate and its byproduct methylmalonic acid can induce a pro-fibrotic phenotype in both epithelial cells and fibroblasts by activating the canonical transforming growth factor-β/Smad pathway [103].

**Figure 5.**
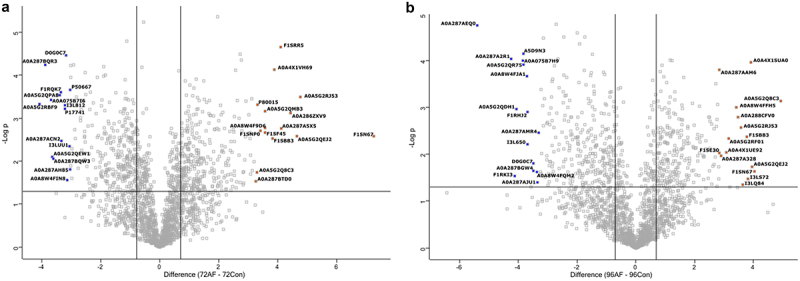
Volcano plots showing the distribution of statistically significant and differentially abundant (SSDA) proteins which have a −log (*p*-value) > 1.3 and difference ± 0.58. (a) *Sus scrofa* infected lung explants with *A. fumigatus* compared to uninfected lung explants at 72 hours and (b) *Sus scrofa* infected lung explants with *A. fumigatus* compared to uninfected lung explants at 96 hours.

The later response to infection elicited many changes in the tissue proteome, supporting the impact of *A. fumigatus* is associated with tissue remodeling and fibrosis (Figure 5a,b) (Table S6). At 72 h post-infection, 346 proteins were increased in abundance and 356 were decreased in abundance. There is further evidence of tissue remodeling and fibrosis with transforming growth factor beta-1-induced transcript 1 protein increased + 7.06 fold, indicating that TGF-β could drive fibrosis following fungal infection. Many components of the extracellular matrix associated with fibrosis were significantly increased in abundance, including fibrillin 1 (+151.93 fold), collagen type IV alpha 2 chain (+26.94), alpha 1 chain (+24.92), alpha 4 chain (+21.14), and alpha 3 chain (+10.67) [104]. There is evidence of an immune response to fungal infection due to the increased abundance of proteins associated with MHC class II signaling, including SLA class II histocompatibility antigen DQ haplotype C alpha chain (+7.04 fold) [105]. MHC class II, DM beta (major histocompatibility complex, class II, DM beta) (+2.83 fold), and ABC-type antigen peptide transporter (TAP2) (+2.71 fold) are involved in antigen processing and presentation and T cell activation [106].

Glutathione-S transferase (−16.87 fold) was decreased in abundance at 72 h and is involved in phase II metabolism and is also an important mediator of normal lung growth [107]. The complement system was again observed to be compromised, with a variety of associated proteins reduced in abundance, including ficolin 2 (−14.64 fold), complement C3 (−1.97 fold), complement C4A (Rodgers blood group) (−2.46 fold), complement factor H (−2.97 fold), complement C5 (−3.42 fold) and complement factor B (C3/C5 convertase) (−6.76 fold). Gene enrichment analysis (Figure S6) highlighted an increase in ECM receptor expression, indicating fibrosis and tissue remodeling, in addition to increased expression of proteins associated with gap junctions, indicating recovery from increased permeability. Gene enrichment analysis also indicated a decrease in the abundance of proteins associated with glyoxylate and dicarboxylate metabolism, glycolysis/gluconeogenesis, and the pentose phosphate pathway, supporting the fact that the infected tissue was less metabolically active than the healthy control (Figure S6).

At 96 h post-infection, 258 proteins were increased in abundance and 308 were decreased (Table S7). Proteins associated with fibrosis, such as fibrillin 1, collagen type IV alpha 1 chain, and alpha 2 chain, were increased by + 16.44, +15.42, and + 11.95, respectively, at 96 h. Collagen type VI alpha 1 chain (+14.16 fold), and alpha 2 chain (+12.37 fold) are associated with pulmonary fibrosis and tissue remodeling, and are elevated in numerous fibrotic conditions [108,109]. In addition, laminin subunit alpha 3, detected at + 14.17 fold and + 13.78 fold at 72 and 96 h, respectively, is increased in pulmonary fibrosis [110]. Proteins associated with immune activity include serine- and arginine-rich splicing factor 3 (+15.10 fold) and expression of which is associated with immune infiltration [111]. SLA class II histocompatibility antigen, DQ haplotype C alpha chain (+6.34 fold) and ABC-type antigen peptide transporter (tap2) (+5.18 fold), MHC class II histocompatibility antigen SLA-DRB1 (+2.43 fold) and SLA-DRA (+2.12) involved in antigen presentation which were also detected at the 72 hours. Mesencephalic astrocyte-derived neurotrophic factor was found to be decreased −19.02 fold, deficiency of this protein in macrophages promoted macrophages to M1 differentiation in lung tissue, contributing to inflammation and aggravated lung injury in mice [112]. Despite this, other protein changes indicate fungal antagonism of the inflammatory response, with a decrease in allograft inflammatory factor 1 (−14.12 fold). AIF1 promotes macrophage activation and regulates immunity by mediating the differentiation and function of dendritic cells [113]. Syntaxin 3 was also decreased by-14.21 fold and is required for the maximal release of IL-1α, IL-1β, and IL-12, and is involved in MMP-9 exocytosis during gelatinase degranulation [114], again indicating that neutrophil activity is impaired by *A. fumigatus*. In addition, copper transport protein ATOX1 was decreased 11.25 fold, deficiency of this protein was shown to reduce recruitment of monocytes/macrophages, and is associated with impaired angiogenesis and wound healing [115]. Gene enrichment analysis (Figure S7) also highlighted increased ECM alterations and proteins associated with focal adhesion, indicating tissue damage. Aspects of the complement cascade are also observed as being compromised in addition to the phagosome, both of which are immune mechanisms known to be inhibited by *A. fumigatus* through the action of enolase and gliotoxin, respectively.

## Discussion

The initial establishment of *A. fumigatus* infection and host adaptation processes have not been fully characterized. Understanding these processes could shed light on the diverse spectrum of infections caused by *A. fumigatus* and may provide insights into how to detect and treat these infections more effectively. A variety of model systems have been developed to characterize the development of *A. fumigatus in vitro* and the results have been useful for understanding how the fungus may interact with pulmonary tissue *in vivo*. Compared to the more commonly used model organisms, pig lungs demonstrate a higher degree of similarity to human lungs, sharing similarities in metabolic composition, overall physiology, anatomy, and immunology [15,116]. The EVPL system has previously been developed to mimic human airways in CF, and has successfully demonstrated clinically realistic biofilm structures [40,117], providing insight into how antibiotic tolerance is affected by growth on a realistic lung substrate [118]. The EVPL model is best suited to study saprophytic or chronic infections such as chronic pulmonary aspergillosis but may not be suitable for studying invasive infections as there are no other tissues to disseminate to. In addition, proteomic signals indicate adaptive immune activation but as the tissue is isolated and non-resident recruitment cannot occur understanding the impact of these signals later in the infection process remains elusive. The timeframe in which the experiments can be conducted is also limited as the tissue cannot be kept for long periods while murine studies can be conducted over a longer timeframe. The EVPL model offers a greater cellular complexity to that observed in epithelial models while demonstrating similar responses including TGF-β production in human primary bronchial epithelial cells following exposure to *A. fumigatus*. [119] Epithelial cell models lack the cellular complexity and cell-cell interaction that were observed in the explant model. Most *in vitro* studies on *A. fumigatus* pathogenesis have focused on one host cell type, but rarely capture the interactions that would occur in a multi-cell system as present in the lung [120].

In this study, the EVPL model was successfully adapted for studying *A. fumigatus* colonization. Alveolar tissue sections were combined with commonly available tissue culture media (1:1 mixture of RPMI and DMEM). This medium was used in the first publications exploring the potential use of post-slaughter pig lung tissue as an *ex vivo* infection model, and was shown to allow long-term culture of pig tissue [37]. Furthermore, tissue culture media has been proposed as an improved medium for clinically predictive antimicrobial susceptibility testing [43]. There is a plethora of more tailored, chemically defined media available that have been designed to mimic human airway secretions in health and diseases, including conditions that predispose individuals to *A. fumigatus* infection, such as CF with various media developed to mimic this condition [121,122] and future work could combine these with EVPL to study *A. fumigatus* in conditions that mimic the host environment found in specific infection contexts. However, in the first exploration of *A. fumigatus* growth and metabolism in EVPL, we elected to use a general-purpose, widely accessible, and cheap growth medium to facilitate model adoption and gain a first look at how this pathogen acts in settings that are more human-like than *in vitro* or mouse models.

Visual inspection of the tissue and agar sections confirmed the ability of the fungus to grow on both the substrates. There was a significant reduction in fungal growth on the tissue explants compared to the agar blocks and this is likely to have occurred as a result of immune antagonism occurring within the tissue as observed in the proteomic results, which would not be present in the agar control. In addition, the increased complexity of the pulmonary tissue could have delayed growth. Proteomic analysis of the fungus indicated an increased abundance of proteins associated with growth and carbohydrate metabolism at 48 h, whereas at 72 and 96 h proteins associated with amino acid metabolism were increased in abundance. Glyceraldehyde-3-phosphate dehydrogenase expression is associated with conidial germination, is expressed on the hyphal surface, and phosphoglycerate kinase is also involved in carbon metabolism, but is also involved in fumigaclavine C biosynthesis [50]. Amino acid metabolism and biosynthesis were more prevalent at 72 h post-infection with aconitate hydratase, mitochondria, 5-methyltetrahydropteroyltriglutamate – homocysteine S-methyltransferase, 4-aminobutyrate aminotransferase, and acetohydroxy-acid reductoisomerase involved in the biosynthesis of lysine, methionine, and glutamate L-isoleucine, respectively, all of which were significantly increased in abundance. Amino acid metabolism and the shikimate pathway have been identified as markers of clinical isolates [123] indicating EVPL induces *A. fumigatus* to behave in a manner similar to that observed in clinical isolates. Malate dehydrogenase detected at 48, 72, and 96 h, is involved in carbon metabolism and is a component of the methylcitrate cycle, an alternative metabolic pathway that plays an important role in the metabolism of propionyl-CoA, a byproduct of amino acids, odd-chain fatty acids, and certain intermediate metabolites [124]. This pathway is the link between the TCA and glyoxylate cycles in fungi and carbon assimilation by *A. fumigatus in vivo* [125]. This pathway is required for fungal survival and pathogenicity, the deletion of which reduces virulence in murine and insect models [126,127].

A range of virulence factors and potential host antagonistic mechanisms were increased in abundance, including fungal allergens at 48 h, such as large ribosomal subunit protein P2 (60S acidic ribosomal protein P2) (AfP2) (allergen Asp f 8) and mitochondrial superoxide dismutase [Mn] (allergen Asp f 6), which may induce the immune response observed within the tissue [51]. These allergens can directly disrupt the integrity of the epithelium and elicit the production of pro-inflammatory cytokines and fibrogenic growth factors. Several cytokines and chemokines have been implicated in the immune response to *Aspergillus* infection [128] and this model may have applications in characterizing these which may shed new insight into their roles in the host response to fungal infection. The ensuing recruitment of immune cells and further leakage of plasma proteins would support the development of a cycle of inflammation, fibrin deposition, and structural changes [129]. Mycotoxin production can also impact the host invasion process, as evidenced by thioredoxin reductase gliT, detected at 96 hours, and methyltransferase psoC, detected at all time points, involved in gliotoxin and pseurotin A production, respectively. Interestingly, fungal enolase, which increased in abundance at all time points, acts as an inhibitor of the human complement cascade [68]. This pathway has been demonstrated to be compromised within the pig proteome, supporting the role of this protein in immune evasion. The results indicate that the fungus is capable of growing on the EVPL tissue and metabolizing the substrate, but the fungus is under stress with evidence of hyphal damage, as evidenced by the formation of the Woronin body and the production of numerous detoxification enzymes.

Proteomic changes in infected EVPL tissues indicate the induction of an immune response. S100-A8 and protein S100-A9 (Calgranulin-B) were increased in abundance at 24 h, which stimulate leukocyte recruitment and induced cytokine secretion [69–71]. There is also evidence of an initial allergic response possibly induced by the expression of allergens, as carbonic anhydrase 4 was also increased in abundance and expressed in IL-5-activated eosinophils. In murine studies, carbonic anhydrase 4 was enhanced following an allergic insult with *A. fumigatus*, resulting in increased airway epithelial cell differentiation, anion exchange, and keratinization [73]. Proteins associated with degranulation decreased in abundance in the infected cohort, including prophenin and tritrpticin precursor (C6), proteinase 3, elastase, and neutrophil expression, indicating specific immune evasion induced by the fungus. At 48 h, there is further evidence of a mounted immune response as importin subunit alpha KPNA3 and KPNA4 were increased in abundance and are essential for TNF-alpha-stimulated NF-kappaB p50/p65 heterodimer translocation into the nucleus [91]. *A. fumigatus* can be detected by Dectin-2 expressed on alveolar macrophages via Syk, which results in Nf-KB activation [130]. MZB1 was also increased in abundance and was expressed on plasmacytoid dendritic cells, which facilitates interferon alpha production following TLR9 stimulation [131].

The first markers of pulmonary fibrosis were detected at 48 hours including indolethylamine N-methyltransferase and nestin [96]. At later time points, there were more prominent markers of pulmonary fibrosis and tissue remodeling, consistent with the situation in immunocompetent mice, where collagen accumulation is reported following infection [132,133]. This may be driven by transforming growth factor beta-1-induced transcript 1 protein increased + 7.06 fold as overexpression of TGF-β is known to induce collagen deposition in mice [134]. Many components of the extracellular matrix increase in abundance during fibrosis and are associated with the establishment of invasive fungal diseases. The increased expression of ECM components could also facilitate the covering of fungal hyphae by the extracellular matrix [135]. This can lead to the formation of aspergillomas where the hyphae are embedded together in this dense extracellular matrix whereas in invasive aspergillosis hyphae are individually engulfed in the matrix [136]. The extracellular matrix coating protects the fungus against host immune effectors as well as antifungal drugs [137]. Protein associated with T cell activation was also found to be increased in abundance, possibly mediated by the NF-KB activation observed previously. This indicates that both innate and adaptive immune responses are active within the tissue. The complement system was compromised, with a variety of associated proteins being reduced in abundance relative to the control. There is further evidence of fibrosis at 96 h post infection with fibrillin 1, collagen type IV, and type VI being significantly increased in abundance [138,139]. Aspects of an adaptive immune response are present, including SLA class II histocompatibility antigen, DQ haplotype C alpha chain, and ABC-type antigen peptide transporter (TAP2) involved in antigen presentation at 96 hours. Prothymosin alpha was decreased in abundance by −42.09 fold potentially occurring as a result of cleavage to its bioactive form, thymosin α1, which has also been shown to enhance maturation of dendritic cells exposed to *A. fumigatus*. This effect was shown to be p38 MAP kinase/NF-κB-dependent and required Toll-like receptor 9 signaling [140]. Some aspects of the immune response appeared to be inhibited by the presence of *A. fumigatus* evidenced by the decreased abundance of allograft inflammatory factor 1 (−14.12 fold). AIF1 promotes macrophage activation and NO production, and regulates immunity by mediating the differentiation and function of dendritic cells [141]. *Leishmania* parasites inhibit AIF1 to effectively evade immune responses and avoid an inflammatory response [142], and it is possible *Aspergillus* may utilize a similar mechanism to protect itself from inflammatory markers. Syntaxin 3 also decreased by-14.21 fold and was involved in degranulation [114], again indicating that neutrophil activity is impaired by *A. fumigatus*.

In a previous study of *P. aeruginosa* infection of bronchioles using EVPL with medium that mimics CF mucus, transcriptomic analysis showed an absence of pig mRNA [117]. This suggests that, in that study, the host tissue was unresponsive to infection. There are two key differences in the present study that may explain this active host response. First, we used alveolar tissue, which provides a much greater number of host cells than bronchiolar epithelium and may contain alveolar immune cells. Second, in the present study, we were able to obtain lungs immediately after slaughter from the abattoir, rather than via a commercial butcher, which necessitates a delay between slaughter and lab use of at least 24–48 hours.

The results presented here highlight the response of porcine lung tissue to *A. fumigatus* infection in a complex biologically relevant model that demonstrates many of the patterns observed in *in vivo* models, as well as clinically. The model demonstrates both innate and adaptive immune responses, tissue remodeling, and fibrosis in response to fungal infection and demonstrates responses observed in allergic and invasive aspergillosis. This study provides insights into the initial host-pathogen interactions and highlights the importance of various metabolic processes for *A. fumigatus* colonization of the host, as well as supporting the role of various virulence factors in the establishment of infection. The tissue response to infection provides information on the importance of various immunological effectors and potential targets for fungal antagonism and demonstrates the role of *A. fumigatus* in the establishment of lung remodeling and fibrosis within the airways. These molecular patterns may provide insight into the initial host-pathogen interactions occurring in the human host and provide molecular targets for therapeutics in the future.

## Data Availability

The mass spectrometry proteomics data were deposited in the ProteomeXchange Consortium via the PRIDE [48]partner repository with the dataset identifier PXD060389. (https://www.ebi.ac.uk/pride/archive/projects/PXD060389).

## Author declaration

This work was presented at the Microbiology Society annual conference in Liverpool by AC on 2 April 2025.

## Abbreviations

CF: cystic fibrosis
LFQ: label-free quantitative
FDR: false discovery rates
SSDA: statistically significant differentially abundant
GO: gene ontology
PCA: principal component analysis
RFC: relative fold change
